# Interest in Weight Loss Methods Among Adults and Its Predictors: Sociodemographic Factors, Anthropometric Parameters, and Physical Activity

**DOI:** 10.34172/ijhpm.8493

**Published:** 2025-06-09

**Authors:** Adrian Lubowiecki-Vikuk, Anna Bartkowiak, Elżbieta Biernat, Adam Kantanista

**Affiliations:** ^1^Institute of Management, SGH Warsaw School of Economics, Warsaw, Poland.; ^2^Institute of Economic Sciences, University of Wroclaw, Wrocław, Poland.; ^3^Institute of International Economic Policy, SGH Warsaw School of Economics, Warsaw, Poland.; ^4^Department of Physical Education and Lifelong Sports, Poznan University of Physical Education, Poznań, Poland.

**Keywords:** Weight Management, Weight Loss, Predictors, Overweight, Obesity, Poland

## Abstract

**Background:**

The study aims to determine adults’ interest in weight loss methods and their predictors such as anthropometric parameters, physical activity, and sociodemographic factors.

**Methods:**

A two-step procedure was adopted. First, anthropometric parameters of 1130 Polish adults were taken, and the body mass index (BMI), the waist-to-hip ratio (WHR), and the body fat (BF) percentage were calculated. Next, the participants completed a questionnaire consisting of questions about their interest in different weight loss methods, questions about physical activity, and metric questions. Interest in six arbitrarily selected weight loss methods was measured using a five-point Likert scale. For analysis ordinal logistic regression and omnibus likelihood ratio tests were used.

**Results:**

The results proved different predictors of adults’ interest in weight loss methods (measured on an ordinal scale). More interest in physical activity and diets as methods of weight loss is observed in underweight individuals (compared to normal body weight status), older, with better economic situation, and higher level of physical activity individuals. In the case of bariatric surgery and liposuction, more interest in this weight loss methods are observed in overweight (compared to normal body weight status), economically well-off participants, and in women (compared to men). More interest of liposuction as a weight loss method is observed in overweight. Interest in dietary supplements to support weight loss (DSSWL) is more in women (compared to men), higher WHR, better economic situation, higher level of education and older individuals. More interest in weight loss drugs is observed in women (compared to men) and economically well-off people.

**Conclusion:**

The key predictors of interest in weight loss methods are body weight status, gender, and economic situation. These vary in importance depending on the type of weight loss method. Healthcare providers should recommend various weight-management strategies, having regard for the level of interest in weight loss methods and their predictors.

## Background

**Key Messages**

**Implications for policy makers**

- Policy-makers need to create opportunities for overweight or obese adults to use appropriate weight loss methods.
- Policy-makers must provide targeted education and training about the risks and benefits of using different weight loss methods.
- Health promotion policies for adults in towns with high rates of overweight and obesity should be based on economic interventionism to develop and implement appropriate tools to shape the population’s healthy eating habits and active lifestyle programmes; consideration should be given to legislation restricting access to weight loss methods with high health risks.

**Implications for the public**

The results of our study are community-oriented and provide a deeper understanding of adults from towns with a high rate of obesity interested in weight loss methods. A high level of interest in the use of different weight loss methods was observed among adults, which can be seen as a positive attitude of people towards weight management. However, before engaging in a weight loss strategy, people need targeted education from healthcare providers to use and avoid appropriate weight loss methods, for example, addiction to physical activity or overuse of dietary supplements. Healthy weight loss methods could be promoted, eg, through a public awareness campaign, depending on local circumstances.

In times of the continuing global epidemic of obesity,^1^ but also socio-cultural standards of attractiveness (ie, slimness for women and muscularity for men)^2^ it is important for people of different ages to maintain a balanced approach to weight management.

 Weight management is a complex concept.^3^ The key concepts within the theory of weight management are weight management behaviours, weight management agency, weight contextual predictors, and weight control.^4^ In the case of weight management behaviours, both healthy populations and those with diagnosed diseases use different methods to lose or maintain weight.^5-7^ There are methods of weight loss involving activities that are considered safe and recommended by healthcare organisations, but some widely applied methods involve disordered behaviour, such as inducing dangerous diets,^8^ vomiting, using laxatives, or smoking.^9^

 Overall, the weight loss methods focus on three weight management strategies: health-oriented (dietary/mindful eating and physical activity), treatment-oriented (drugs and dietary supplements) and invasive (bariatric surgery and liposuction).^5^ The first weight management strategy relates to weight loss methods based on behaviour change. These include exercise or physical activity^10-12^ and following a balanced diet^13^ but incorporating exercise or diet into the daily routine requires effort. The second weight management strategy uses dietary supplements to support weight loss (DSSWL) and drugs. DSSWLs are presented as requiring fewer changes in everyday activities. They are strongly promoted in advertisements with claims of efficacy and are readily available without a prescription.^14^ Interest in dietary supplements is found in different regions around the world.^15^ This weight management strategy requires much more money from patients (much more than exercise), which significantly limits its availability.^16^ In addition, Xing et al^17^ indicated that the use of DSSWL or weight loss drugs may have side effects, there is consumer scepticism about their efficacy, and that they are a short-term solution to weight control.

 Finally, the third weight management strategy involves invasive methods. Interest in surgical procedures has increased in recent decades among men and women. Liposuction was one of the most popular.^18-20^ According to the American Society of Plastic Surgeons,^21^ more than 700 000 surgical procedures include liposuction. In addition to liposuction, invasive weight loss methods involve bariatric surgery, which has seen a significant increase in recent decades.^22^ Bariatric surgery is the most effective option, with a 20% to 40% reduction in body weight.^23^ It is usually performed on adults who declare less physical activity, more fast food and fat consumption, and less dietary restraint.^24^

 People decide to employ weight loss methods very often due to the internalization of social standards of attractiveness and concerns about body image.^25,26^ Jávo et al^18^ indicated that overweight women with eating problems who have a poor evaluation of their appearance have a strong interest in liposuction. Moreover, socio-environmental and relational individual factors such as education,^27^ marital status,^28-30^ and the body mass index (BMI),^31^ affect the level of this interest. Medical procedures and daily behaviour aimed at weight loss are extremely popular topics in women’s (and men’s) fashion and beauty magazines. Cosmetic surgery is also an aspect of many popular reality-based television programs.^32^ It may also affect the level of interest in weight loss methods among people who do not need behavioural, pharmacological, or invasive intervention.

 In reviewing the literature, we found that little is known about the factors that determine the choice of different weight loss methods among people living in regions with high obesity rates. In addition to socio-demographic factors, the contribution of anthropometric factors can be seen, which we highlight in our article. In addition, we have emphasized the levels of physical activity rather than the total amount of physical activity, which gives a better understanding of the choice of different weight loss methods.

 In practice, weight management strategies enable the appropriate, health-promoting weight control programmes to be devised in a community setting, especially in high-prevalence obesity settings. The study aimed to examine interest in weight loss methods among Poles from towns with high obesity rates. Furthermore, our aim was to evaluate differences in interest in these methods according to predictors such as sociodemographic characteristics, anthropometric parameters, and levels of physical activity.

## Methods

###  Participants

 Adult participants were recruited through a public invitation, which consisted of announcements displayed on notice pillars belonging to the town hall. There were 1130 adult participants in the study (mean _(age)_ = 44.4 years, standard deviation [SD] = 15.6 years), 628 women (mean _(age)_ = 43.3 years, SD = 15.3 years) and 502 men (mean _(age)_ = 45.6 years, SD = 15.9 years). All participants were residents of Świętochłowice – a Polish town in the Silesian Voivodship with one of the three highest rates of adult obesity in Poland.^33^ The characteristics of the respondents are described in Tables S1–S6 (Supplementary file 1).

 The study was conducted by trained and supervised interviewers who had a degree in Pharmacy, according to a predetermined plan, just before the COVID-19 pandemic outbreak.

 It was a two-step study. In the first step, weight and height were measured, as well as waist and hip circumference, and then BMI and waist-to-hip ratio (WHR) were calculated, and body fat (BF) status was assessed. Maintaining the comfort of the participants, the anthropometric measurements were taken in a designated and secluded area. In the second step, participants were asked to fill out a paper questionnaire about their level of physical activity, sociodemographic characteristics, and interest in weight loss methods. Of the 1130 surveys, the majority of questions were fully answered by the respondents (from 1088 to 1118). The exception is the questionnaire assessing the level of physical activity, where the lack of complete answers resulted in the possibility of analysis of results from 822 to 829 individuals. Before the questionnaire interview, respondents were informed about the purpose of the survey and that they could discontinue their participation at any time without any consequences. Participation in this study was completely voluntary, excluding only individuals under 18 years old, and people with physical or intellectual disabilities that prevented them from taking part in particular assessments.

###  Measurements

####  Interest in Weight Loss Methods

 Three weight management strategies were selected for the study, with six weight loss methods assigned to them: (1) health-oriented (taking up physical activity, weight loss diet), (2) treatment-oriented (using DSSWL and/or weight loss drugs), and (3) invasive (bariatric surgery or liposuction). The respondents in the questionnaire stated to what extent they were interested in each weight loss method using a five-point Likert scale (with 1 – denoting “definitely not,” 2 – “rather not,” 3 – “neutral,” 4 – “yes,” and 5 – “definitely yes”), with answer categories treated as continuous variables.^34^

####  Sociodemographic Factors

 Sociodemographic variables were collected in a questionnaire where gender (man, woman), age (18–39, 40–59, 60 and over), marital status (married or unmarried), education level (primary, vocational, secondary, higher) and professional activity (professionals, technicians and associate professionals, service and sales workers, crafts and related trade workers, plant and machine operators and assemblers, elementary occupations, and non-employees) were included. In addition, respondents were asked about their subjective assessment of their economic situation (three responses: “poor,” “good,” or “hard to say”).

####  Level of Physical Activity

 The Polish version of the International Physical Activity Questionnaire-Short Form (IPAQ-SF)^35^ was used to assess the level of adult physical activity. The respondents filled in the IPAQ-SF by themselves as recommended by Biernat et al.^35^ The results were presented as estimated energy expenditure as a metabolic equivalent (MET). Participants were classified according to physical activity level, as “low” (<600 MET-min week^-1^), “moderate” (600–2999 MET-min week^-1^) and “high” (≥3000 MET-min week^-1^). The questionnaire is commonly used and features good psychometric indicators.

####  Anthropometric Measurements

 The weight of the participants was measured using the Omron scale. Height was measured with an anthropometer. BMI (kg/m^2^) was calculated using the body height and weight of the participants. BMI norms were applied according to Weir and Jan.^36^ Waist circumferences and hip circumferences were measured to calculate the WHR.^37^

 An Omron Body Fat Analyzer model HBF-360 (Omron Healthcare, Inc., Vernon Hills, IL, USA) was used to measure BF. According to Malavolti et al,^38^ this is a valid, non-invasive, inexpensive method. The most frequently used BF (status) cutoff points for defining obesity (25% in men, 35% in women) were applied.^39,40^

###  Data Analysis

 First, polychoric correlations and correspondence analysis were used to determine the relationship between dependent variables. Kendall’s tau-b or Cramer’s V test and chi-square test were used to determine whether there was a correlation between variables. Next, for independent variables with a significant relationship with the dependent variable and a non-significant relationship with each other, one of the generalized linear models, ordinal logistic regression analyses, was conducted. The odds ratio (OR) with the 95% confidence interval (CI) were calculated. To determine the significant effect on the dependent variable, omnibus likelihood ratio tests were used. The threshold for statistical significance for all tests was set at 0.05.

 All calculations were performed using Jamovi version 2.3. Violin plots were used to illustrate the results, adding a rotated kernel density plot on each side, making it much easier to compare observations.^41^

## Results

 Health-oriented weight loss methods, namely undertaking physical activity and following a weight loss diet (for which the responses were “rather yes” (64%) and “definitely yes” (61%), are the most popular weight loss methods (Tables S1–S2). In particular, a significant variation was observed among respondents who showed an interest in treatment-oriented methods (53% for the responses “rather yes” and “definitely yes” for DSSWL and 35.7% for “definitely not” and “rather not” for weight loss drugs) (Tables S5–S6). A substantial percentage of respondents did not have a strong opinion regarding their interest in invasive methods for weight loss (bariatric surgery – 30%, liposuction 28%), while many respondents said that they were not interested in them (Tables S3–S4).

 Using polychoric correlation, a strong relationship was obtained between physical activity and weight loss diet (0.894), bariatric surgery and liposuction (0.918), DSSWL and weight loss drugs (0.821).

 Using the correspondence analysis (Figure S1, Supplementary file 2) and Gaussian graphical method, three groups of similar variables were obtained: physical activity and weight loss diet, bariatric surgery, liposuction, and DSSWL, and weight loss drugs.

 When analysing the associations between different weight loss methods, we found that there was a strong correlation between physical activity and weight loss diets, and between bariatric surgery, DSSWL, and weight loss drugs. There is a weak correlation between physical activity and bariatric surgery and liposuction, and between a weight loss diet and bariatric surgery and liposuction.

 Based on the relationships between dependent variables and predictors (Table S7, Supplementary file 3) a selection of variables was applied to the model using the correlation coefficient method. Next, ordinal logistic regression was used for each dependent variable. Not all independent variables were found to be statistically significant, so they were removed using the backward stepwise selection from further analysis. The significant predictors for each independent variable are shown in Table S8 (Supplementary file 3).

 The following factors had a significant impact on interest in physical activity: level of physical activity, BMI, age, and economic situation. Interest in diet is further influenced by education level instead of economic situation. Interest in bariatric surgery and liposuction was linked to BMI, economic situation, and gender. Interest in DSSWL was affected by WHR, age, economic situation, education level, and gender. Interest in weight loss drugs was linked to economic situation, gender, and professional activity.

###  Interest in Undertaking Physical Activity

 Level of physical activity is expected to influence interest in physical activity as a means of weight loss; 1.5 (OR = 1.46, 95% CI 0.97, 2.18) times more interest is shown by those with moderate activity, and almost three times (OR = 2.75, 95% CI 1.76, 4.31) more interest by those with high levels of daily physical activity than those with low levels. Underweight people are five times (OR = 4.97, 95% CI 2.00, 15.26) more interested in physical activity as a weight loss method compared to people of normal weight. Overweight people, on the other hand, are approximately 35% (OR = 0.64, 95% CI 0.47, 0.86) less interested in this method of losing weight compared to people with correct BMI.

 The level of interest in physical activity as a mean of weight loss decreases with age, by around 65% for people aged 60 and over; (OR = 0.35 95% CI 0.24, 0.50) compared to young people (Table S9). The better the economic situation, the significantly higher the level of interest in physical activity, up to 10 (OR = 10.32, 95% CI 6.15, 17.54) times higher for those with a good economic situation compared to those with a poor economic situation.

**Figure 1:**
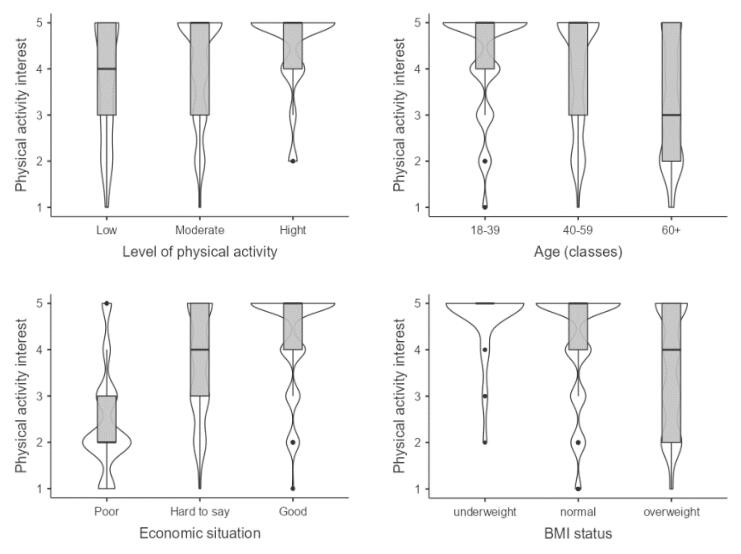
Violin and Box Plots for Physical Activity Splint by Significant Variables. *Notes:* Violin plot – illustrates the rotated distribution of the density of responses on each side. Box plot is drawn in the center of the violin plot, the box represents the interquartile range, covering the middle 50% of the data, and the whiskers (thin vertical lines going from the horizontal edges of the box) extend to the minimum and maximum observed values within a reasonable range. The thick horizontal line in the center of the box is the median. The dots are outliers; for physical activity interest: 1 – definitely not, 2 – rather not, 3 – no opinion, 4 – rather yes, 5 – definitely yes. Abbreviation: BMI, body mass index.

 People who are underweight and of normal weight are more willing to engage in physical activity and are more homogeneous than those who are overweight (Figure 1). Interest in physical activity for weight loss purposes decreases with age, and the population becomes more diverse in this aspect. People with a poor economic situation are generally not interested (63%) in physical activity at all. In contrast, almost all people in good situations are interested (84%) in this way of losing weight.

###  Interest in Weight Loss Diets

 Level of physical activity is expected to influence interest in diet as a means of weight loss; 1.6 (OR = 1.61, 95% CI 1.03, 2.50) times more for those with high levels of daily physical activity than those with low levels. Underweight people are almost five times more interested in a weight loss diet (OR = 4.76, 95% CI 2.00, 13.42) compared to people of normal weight. On the other hand, respondents with obesity are approximately 40% (OR = 0.59, 95% CI 0.38, 0.90) less interested in this method compared to people with normal BF. People aged 40–59 are the same (OR = 1.09 95% CI 0.78, 1.52) and people of 60 and over are approximately 40% (OR = 0.63 95% CI 0.44, 0.90) less interested in diet compared to young people. The better the economic situation, the significantly higher the level of interest in diet, up to almost five (OR = 4.69, 95% CI 2.68, 8.30) times higher for those with a good compared to those with a poor economic situation. The higher the level of education, the greater the interest in a weight loss diet method. People with higher education are up to six times (OR = 6.18, 95% CI 2.68, 14.28) more interested in this method. However, in the two last cases, caution should be exercised with the interpretation of the multiplicity here due to the large CI (Table S10).

**Figure 2:**
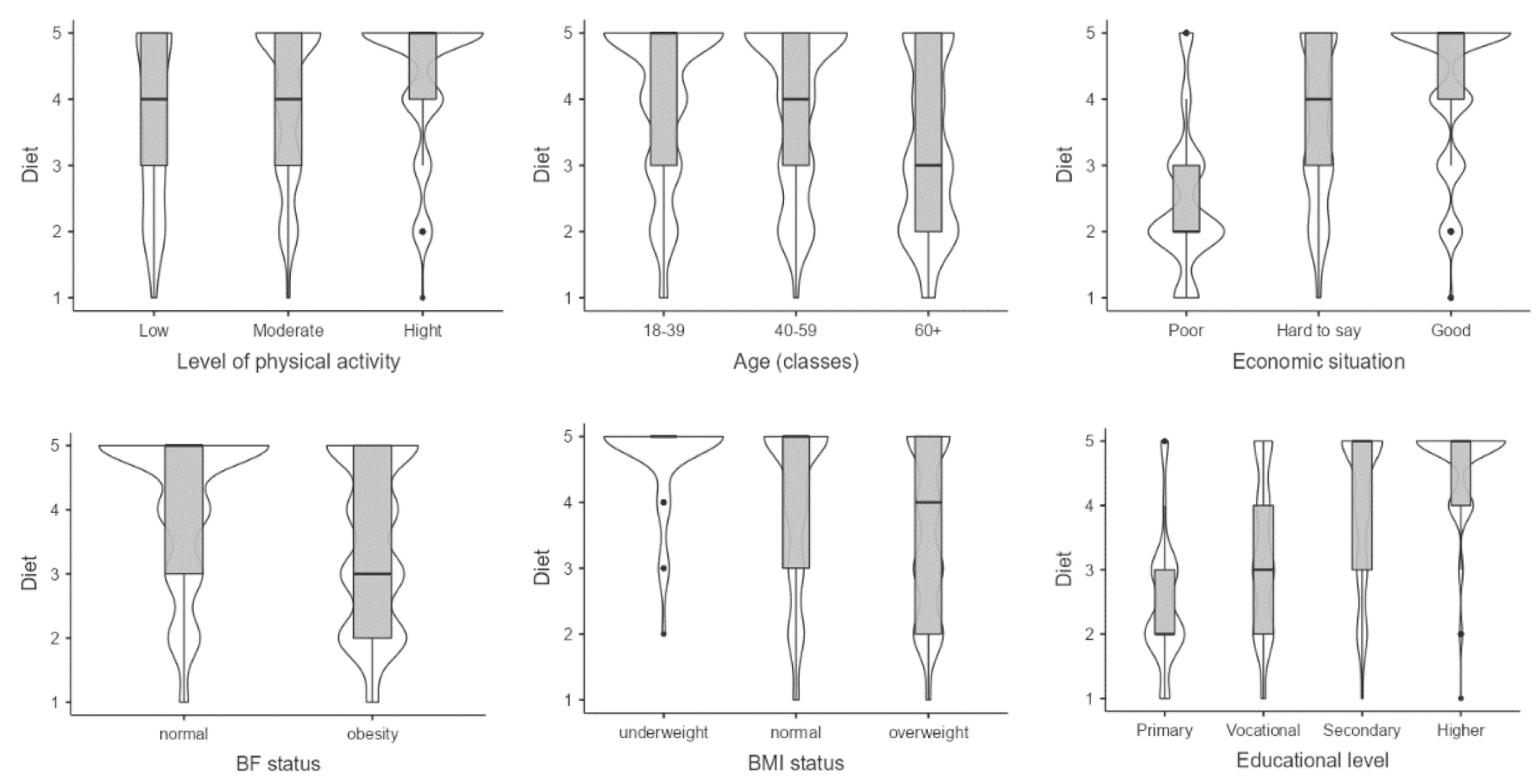
Violin and Box Plots for Weight Loss Diet Splint by Significant Variables. Notes: See note for Figure 1; for weight-loss diet interest: 1 – definitely not, 2 – rather not, 3 – no opinion, 4 – rather yes, 5 – definitely yes. Abbreviations: BMI, body mass index; BF, body fat.

 People who are active to a low or medium degree are similarly interested in a weight loss diet, while very active people are much more interested in it (Figure 2). Respondents who are underweight and have normal BF are more willing follow a dieting plan than those who are overweight/obese. Respondents under 60 are similarly (older people slightly less so) interested in weight loss diets, whereas older people are much less interested. The better the economic situation, the significantly greater the interest in diet. In contrast, almost all people in good situations are interested (84%) in this way of losing weight. The same is observed with education level.

###  Interest in Bariatric Surgery

 Overweight people are approximately 1.5 (OR = 1.47, 95% CI 1.17, 1.85) times more interested in this method of losing weight compared to people with the correct BMI. Economically well-off people are almost three (OR = 2.8, 95% CI 2.06, 3.83) times more interested in bariatric surgery than poorer people, and women are 1.8 (OR = 1.82, 95% CI 1.46, 2.26) times more interested in this surgery than men (Table S11).

**Figure 3:**
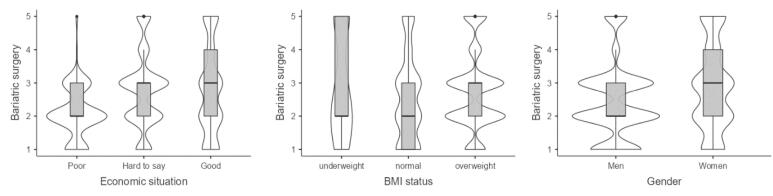
Violin and Box Plots for Bariatric Surgery Splint by Significant Variables. Notes: See note for Figure 1; for bariatric surgery interest: 1 – definitely not, 2 – rather not, 3 – no opinion, 4 – rather yes, 5 – definitely yes. Abbreviation: BMI, body mass index.

 Respondents who are underweight are more willing to have bariatric surgery, and people who are overweight are a little more willing than those who have normal BMI (Figure 3). The better the economic situation, the greater the interest in bariatric surgery. Women are more interested in this way of losing weight than men.

###  Interest in Liposuction

 Overweight people are approximately 1.4 (OR = 1.35, 95% CI 1.08, 1.70) times more interested in this method of losing weight compared to people with the correct BMI. Economically well-off people are more than three (OR = 3.18, 95% CI 2.33, 4.35) times more interested in liposuction than poorer people, and women are two (OR = 2.13, 95% CI 1.71, 2.65) times more interested this method of losing weight than men (Table S12).

**Figure 4:**
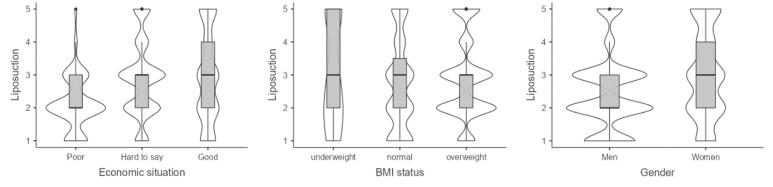
Violin and Box Plots for Liposuction Splint by Significant Variables. Notes: See note for Figure 1; for liposuction interest: 1 – definitely not, 2 – rather not, 3 – no opinion, 4 – rather yes, 5 – definitely yes. Abbreviation: BMI, body mass index.

 Respondents who are underweight are more willing to have liposuction, and people who are overweight are slightly less willing than those who have normal BMI (Figure 4). The better the economic situation, the greater the interest in liposuction. Women are more interested in this way of losing weight than men.

###  Interest in Dietary Supplements to Support Weight Loss

 Women are 2.5 (OR = 2.49, 95% CI 1.98, 3.14) times more interested in DSSWL than men. Respondents with abdominal obesity compared to people with normal WHR are approximately 1.5 (OR = 1.51 95% CI 1.07, 2.14) times more interested in this method. People aged 40–59 1.4 (OR = 1.37 95% CI 1.05, 1.79) times more interested in food supplements, and people 60+ are approximately 40% (OR = 1.37 95% CI 1.05, 1.79) more interested in food supplements compared to people under 40. The better the economic situation, the higher the level of interest in DSSWL, up to almost 4.5 (OR = 4.49, 95% CI 3.07, 6.60) times higher for those with a good compared to those with a poor economic situation. The higher the level of education, the greater the interest in DSSWL. People with higher education are up to 4.6 times (OR = 4.61, 95% CI 2.54, 8.35) more interested in this method. However, caution should be exercised with the interpretation of the multiplicity here due to the large CI (Table S13).

**Figure 5:**
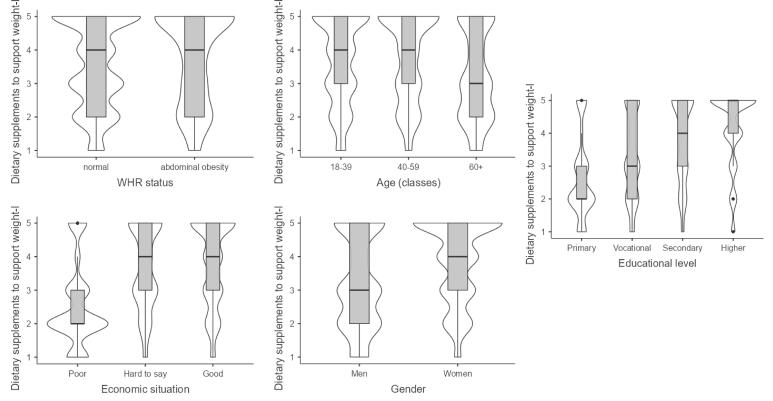
Violin and Box Plots for Dietary Supplements to Support Weight Loss Splint by Significant Variables. Notes: See note for Figure 1; for dietary supplements to support weight-loss: 1 – definitely not, 2 – rather not, 3 – no opinion, 4 – rather yes, 5 – definitely yes. Abbreviation: WHR, waist-to-hip ratio.

 Respondents who have abdominal obesity are a little more willing to use DSSWL than those who have normal WHR (Figure 5). Respondents under 60 are similarly (slightly less so in older people) interested in DSSWL, whereas older people are much less interested. People with a poor economic situation have a significantly lower level of interest in DSSWL. In contrast, almost all people in a “good” economic situation are interested (61% compared with 16% for poor people) in this way of losing weight. The same is observed with education level. The higher the level of education, the greater the interest in this method of losing weight.

###  Interest in Weight Loss Drugs

 Only plant and machine operators and assemblers are statistically significantly less (approximately 40%) interested in weight loss drugs compared to professionals. Economically well-off people are more than five (OR = 5.15, 95% CI 3.60, 7.38) times more interested in weight loss drugs than poorer people, and women are more than two (OR = 2.31, 95% CI 1.79, 2.99) times more interested in this method than men (Table S14).

**Figure 6:**
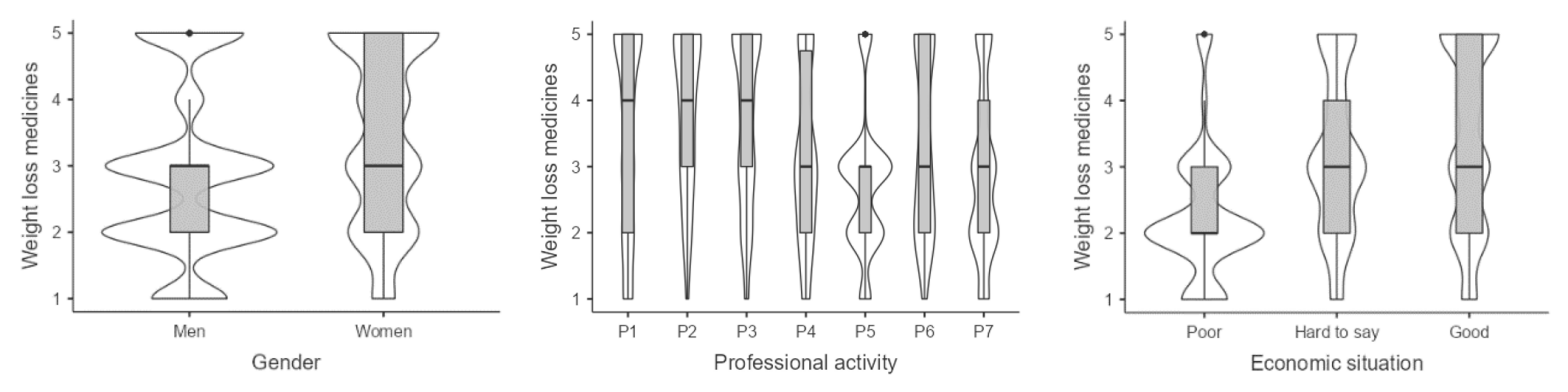
Violin and Box Plots for Weight Loss Drugs Splint by Significant Variables. Notes: See note for Figure 1; for weight-loss drugs: 1 – definitely not, 2 – rather not, 3 – no opinion, 4 – rather yes, 5 – definitely yes; professional activity: P1 – professionals, P2 – technicians and associate professionals, P3 – service and sales workers, P4 – craft and related trades workers, P5 – plant and machine operators and assemblers, P6 – elementary occupations, P7 – non-employees.

 Plant and machine operators and assemblers are least interested in this method of weight loss, and technicians and associate professionals, and service and sales workers are the most interested (Figure 6). The better the economic situation, the greater the interest in weight loss drugs. Women are more interested in this way of losing weight than men.

## Discussion

 The purpose of this study was to examine adult Poles’ interest in weight loss methods in relation to sociodemographic background, anthropometric parameters, and lifestyle factors, such as level of physical activity. The following weight loss methods were considered: health-oriented (physical activity, weight loss diet), treatment-oriented (using DSSWL or weight loss drugs) and invasive weight loss method (undergoing bariatric surgery or liposuction).

###  Physical Activity and Weight Loss Diet

 The results show that approximately half of the respondents expressed an interest in physical activity as a weight loss method. This is not surprising, since exercise – as an integral part of any weight loss program – is recommended by major public health organizations, such as the American College of Sports Medicine and American Diabetes Association.^42^ According to studies^43,44^ (not all^45,46^) the best results in weight loss are obtained through exercise alone. They are obtained by combining exercise with dietary restrictions.^47^

 What matters for long-term weight loss success is individuals’ general knowledge of how to improve physical fitness and health.^48^ One other fact, highlighted by Skrok et al,^49^ is worth noting, namely that participation in sports and recreational activities is strong motivation to continue. Thus, it is possible that physically active people somewhat habitually (but also intentionally) choose to exercise to reduce weight. Piątkowska and Biernat^50^ confirm that habits can be positively related to physical activity and can partially regulate it.

 Previous literature^51^ well documented the fact that highly educated people in particular show favourable eating behaviours. Similarly, in our study, respondents were six times more likely to be interested in dieting as a weight loss method than those with primary education.

 Another factor determining interest in physical activity and dieting as weight loss methods is age. On the one hand, this shows that middle-aged and older people are passive about physical activity and following a diet, and on the other hand it shows that younger people are active. The passivity of the elderly may be due to various factors, such as physical (such as loss of muscle strength, loss of muscle mass, balance, and flexibility problems) or psychological factors (depression, apathy, fear of injury).^52^ Many elderly people suffer from various ailments, such as joint pain, back problems, chronic illnesses, or other conditions that can hinder exercise.^53^ Social isolation and lack of support and companionship are not conducive to motivation to undertake physical activity^53^ and diet.^54^ With age, there may also be changes in appetite and food preferences – which can result in poor diet quality and worsening unhealthy eating habits.^55^ Finally, some older people may not realize the benefits of taking physical activity and eating healthily regularly. Researchers have long urged that education and health awareness are key to taking control of one’s health.^56^

 The greater interest in physical activity and dieting among 18–39-year-olds, in turn, may be a result of the presence of a cultural pattern in Western societies, placing great importance on appearance.^57^ It turns out that between 1/3 and 2/3 of adults in Western populations (regardless of their BMI) are at any given time on a diet aimed at either losing weight or maintaining weight^58^ – and they are mostly young people.^59^ Since the 1990s, this trend has been gaining momentum.^60^ Some of those losing weight claim to be on a diet practically all the time. Weight loss plans are also started by people who objectively have no reason to do so (whose body weight in relation to height is within the norm),^60^ especially women. Not surprisingly, Putterman and Linden^59^ believe that health-promoting strategies should distinguish between those who are on a diet to improve their appearance and those who want to improve their health.

 The results also show that physical activity and dieting are five times more likely to be mentioned by underweight people (relative to people of normal weight). Because of – noted in the previous literature – the higher risk of eating disorders (pathological eating behaviours such as starvation, and unbalanced diets)^8,9,13,18^ and other health problems, this group deserves special attention. Among our respondents – as among those analysed by other researchers^60^ – this group is mostly women (61%). Researchers warn that slimness, which in Western societies is one of the most important cultural markers (not only of female beauty but sometimes of women’s worth),^61^ may lead to an obsessive drive to lose weight and to a distorted perception of one’s own body.^62^ This in turn leads to an above-average interest in exercise and diet.^63^

 When it comes to overweight respondents, the frequency of diet use (relative to those with a normal BMI) is 1.2 times higher. However, no such correlation is found for physical activity. The reason for this may be an aversion to physical activity (coupled with the associated exertion and fear, as mentioned above) or a lack of knowledge about the use of safe and effective exercises, which other researchers also point out.^50^ An important problem may also be a psychological barrier (related to self-perception and embarrassment during exercise), considered to be the main obstacle to adults with obesity undertaking physical activity.^64^ In such a context, choosing a diet (one that is more accessible, easier to understand and provides privacy) is a simpler and faster way to achieve results, especially if one does not have to spend much time on regular workouts.^65^ Obese people succumb to social pressure and the culture of weight loss,^66^ in which dieting is promoted as a quick way to lose weight, while messages in the media suggest that it is the only effective solution.

 In our study, it was also observed that people with obesity are approximately 40% less interested in dieting compared to those with normal BF – which is surprising. They may have plans to engage in a more radical method of weight loss, and therefore consider dieting as a less effective method, outdated at this stage. Our further results indicate, obese people are less interested in bariatric surgery and liposuction than those with normal BMI and WHR. Thus, another reason for the lack of interest in dieting is that people with obesity do not consider it a problem, or a chronic disease,^67^ and therefore may not see the need to seek appropriate treatment. Polish medical associations emphasise that obesity in Poland is diagnosed very late and that it is the most neglected disease of the 21st century.^68^

 Our analysis shows that the better the economic situation of the respondents, the higher their manifested interest in physical activity and diet compared to those with poor economic situations. It has been proven that people with higher socioeconomic status tend to lead healthier lifestyles, which include regular physical activity and diet.^69^ It should also be remembered that diets (in the form of prepared meals) are not cheap, so a better economic situation promotes greater interest in them.

###  Bariatric Surgery and Liposuction

 Fourteen percent of Polish respondents are “definitively” interested in bariatrics, and 16% in liposuction. Interest in these methods may be interrelated because both bariatrics and liposuction are treated as methods that offer some certainty of achieving significant weight loss in a short time.^70^ We suppose that our respondents see them as a faster solution that helps avoid the lengthy process of diet and exercise.

 It is interesting to note that bariatrics and liposuction are more often mentioned by underweight and overweight people. While there is some justification for overweight people, the interest in these methods among underweight people is an alarming sign. After all, bariatric surgery is recommended for people with severe obesity (with a BMI above 40), when other methods do not work.^71^ This again reveals the strong effect of the cultural pattern of Western societies regarding external appearance. This conjecture is indirectly documented by the fact that women are indeed more likely to be interested in bariatrics and liposuction than men, that is, almost two times more often for bariatrics and more than two times for liposuction – as also indicated by other studies.^72^

 Our findings are confirmed by the fact that bariatric surgery is more likely to interest people with normal BMI and WHR than those with obesity. The same is true of liposuction, as people with normal BF and normal WHR are more likely to be interested in it than those with obesity. Again, this suggests inadequate awareness among obese respondents. According to a study commissioned by the American Society for Metabolic and Bariatric Surgery and NORC at the University of Chicago in 2016,^71^ although there is growing concern among the public about the dangers of obesity, only one in three people with obesity said they had ever talked to a doctor about their weight. In addition, only 12% of those with severe obesity said their doctor had ever suggested that they consider bariatric surgery. It is therefore necessary to continue efforts to educate both the public and health professionals about the treatment of obesity with invasive weight loss methods. We know that fear, misconceptions, and prejudices may persist among patients.^73^

 An important determinant of interest in bariatrics and liposuction is socioeconomic status. Namely, people with “good” economic status are almost three times more likely to mention bariatric surgery and more than three times more likely to mention liposuction than people with lower economic status. Interest in bariatrics also increases slightly with education level, as does liposuction. In Poland, liposuction is not a procedure reimbursed by the National Health Fund. Bariatric surgeries are reimbursed, but the lack of specialist doctors results in a wait of several months for surgery.^68^ Actual access is therefore still a challenge, and for this reason these procedures are performed in both domestic and foreign commercial hospitals or clinics,^19^ where the economic status of patients is important.

 Our analysis shows that greater interest in invasive weight loss methods is found among those under sixty than among those over that age. The reason may be the concern that these methods are dangerous and may pose risks related to the general health of the elderly, with anaesthesia during surgery, with the length of recovery and complications especially with existing diseases.^74^ However, the prospective Teen-Longitudinal Assessment of Bariatric Surgery^75^ study proves that metabolic surgery is safe and effective, and bariatric surgery is the most effective treatment to date for people with moderate and severe obesity.^76^

###  Dietary Supplements to Support Weight Loss and Weight Loss Drugs

 Our results confirm that interest in DSSWL and weight loss drugs are interrelated. DSSWL (a mixture of vitamins, minerals, herbs, or other ingredients that support the body without directly affecting weight loss) are a method “definitely” considered by 38% of respondents, while drugs (active substances that have been clinically tested and have proven effects on metabolic processes, appetite suppression, or fat absorption) are mentioned by 27% of individuals. The larger percentage using DSSWL is a result of easier access to them – they can be purchased without going through a doctor, and cost less than drugs. At the same time, women are more likely to take them than men. This is also indicated by other studies.^77^ The situation is similar in the case of drugs. This confirms earlier observations about the tendency to achieve a certain standard of beauty, including a slim figure^66^ through alternative means to diet.^78^

 One other fact worth noting is that these methods are less often used by older people (supplements – 19%, drugs – 20%). This may indirectly confirm our earlier conclusions. Firstly, such weight loss methods are mainly used by young people, especially women, who are concerned about their appearance and want immediate results.^15,79^ Secondly, cooperation with health professionals on the issue of combating obesity (education and possible pharmacological treatment) is still unsatisfactory in Poland. Meanwhile, according to a study by Rychlik et al^80^ in Poles over sixty years of age, 55.3% of men and 40.1% of women were overweight, and 20.3% of men and 21.7% of women were obese (mostly abdominal obesity).

 In only one case, that is respondents with abdominal obesity, is there a greater interest in DSSWL, which are most likely to be taken on their initiative, without knowledge of the credibility and reliability of the information.^81^ It is worth mentioning here the growing market of supplements for which no weight loss effect has been confirmed. The interest in supplements may stem from the fact that people with abdominal obesity—those who are subjected to greater social pressure, often stigmatized, and discriminated against^82^—manifest an increased tendency to experiment with various means, to seek easy and quick solutions, such as dietary supplements. Unfortunately, the use of some of them may have side effects, causing, for example, liver damage, gastrointestinal disorders, increased risk of pancreatitis, thyroid disorders.^83^ People with abdominal obesity, on the other hand, are less likely to point to drugs that must be recommended by doctors as part of a weight loss plan. Those most interested in weight loss drugs are those with BMI below the norm and normal body weight – which is cause for concern. The weight loss industry targets a wide range of people, including those with lower body weight, convincing them that the use of weight loss drugs is key to achieving the ideal figure.^66^ However, this ideal image of a slim body is far from real, making it appear manipulative.^84^

 Our results show a correlation between the use of these two methods and socioeconomic status. Previous literature has consistently shown that supplement consumption in adults increases with income and education.^77,85^ Similarly, in the surveyed group, the better the economic situation, the higher the level of interest in DSSWL and weight loss drugs. In addition, the better educated the respondents, the more often they are interested in DSSWL.^15,86^ Interest in weight loss drugs increases slightly with education level. People with higher education may be likely more aware of the health benefits of various nutrients and dietary supplements. According to their status, they have more financial resources, which allows them to invest in health products. They may also have easier access to health professionals who can recommend specific dietary supplements or drugs to them.

###  Strengths and Limitations

 This study has several limitations. First, it is a cross-sectional survey study, so a cause-and-effect relationship cannot be established. Despite limitations, such as subjectivity, the influence of social expectations, or the varying ability of respondents to recall past events, the questionnaire allows for conducting a population study. Secondly, the results cannot be generalized to the entire Polish population because the study was conducted among residents of Świętochłowice – a town in the Silesian Voivodeship with one of the three highest obesity rates among adults in Poland. Moreover, only volunteer participants took part in the study. However, this study has significant strengths. Specifically, in addition to confirming previous findings that BMI, gender, and economic status are the main predictors of weight loss methods, it highlights correlations between the chosen methods and how different body weight status categories of respondents influence the choice of a particular method. This represents a new aspect and provides an important contribution to understanding awareness and preferences regarding weight loss methods and their determinants. Moreover, to our knowledge, this is the first study in Poland on various weight loss methods and their predictors conducted on a relatively large sample. Obesity was assessed using objective measures, and other body mass indices (BMI, WHR) were also calculated.

 Further research is needed, both quantitative and qualitative (or mixed method), for example using focus groups or individual in-depth interviews focused on a specific weight loss method. It is important that research is conducted in other regions with high rates of obesity, considering cultural backgrounds which could allow generalization of the obtained results. Key questions to be answered include (1) What is the impact of cultural factors on interest in and ultimate choice of a particular weight loss method? and (2) What are the differences and similarities in interest and final choice of weight loss methods due to life cycle effects?

## Conclusions

 Relatively high interest in health-oriented and treatment-oriented, but also in invasive weight loss methods, especially in women, was observed. BMI, gender, and economic situation were the most common factors determining interest in different methods of weight loss. Our research results indicate that the sports and fitness market, as well as the pharmaceutical and medical markets (including plastic surgery), are likely to be of significant interest to adults living in cities in one of the three regions with the highest rates of adult obesity in Poland. We are aware that local conditions (including cultural and economic factors, climate conditions, access to healthcare, lifestyle, preferences of local communities, level of education and health awareness, and others) can be crucial in the choice of weight loss methods and may vary these choices regionally. It seems that people with different body weight status and different levels of sociodemographic factors may use the products and services offered on the market. On the other hand, the great interest in various methods of weight loss requires education from the state regarding, above all, the risks resulting from excessive physical activity, the use of unproven diets, consuming questionable quality DSSWL or weight loss drugs, and the medical consequences of bariatric surgery.

## Acknowledgements

 The authors express their gratitude to Dominika Mucha (MPharm, Ph.D.) for her support in collecting the data.

## Ethical issues

 The study protocol was approved by the Local Bioethical Committee of the Karol Marcinkowski University of Medical Sciences (399/18).

## Conflicts of interest

 Authors declare that they have no conflicts of interest.

## Supplementary files


Supplementary file 1. Respondents’ Characteristics (including Tables S1-S6).

Supplementary file 2 contains Figure S1.

Supplementary file 3. Results (including Tables S7-S14).
