# Supplementary material for: Interest in Weight Loss Methods Among Adults and Its Predictors: Sociodemographic Factors, Anthropometric Parameters, and Physical Activity
Source: Int J Health Policy Manag. 2025 Jun 9;14:8493. doi: 10.34172/ijhpm.8493 (PMC12337211; doi:10.34172/ijhpm.8493)
Supplement: Supplementary file 1 — Respondents’ Characteristics (including Tables S1-S6). [file ijhpm-14-8493-s001.pdf]

Article title: Interest in Weight Loss Methods Among Adults and Its Predictors: Sociodemographic Factors, Anthropometric Parameters, and Physical Activity

Journal name: International Journal of Health Policy and Management (IJHPM)

Authors' information: Adrian Lubowiecki-Vikuk[1]*, Anna Bartkowiak[2], Elżbieta Biernat[3], Adam Kantanista[4]

[1]Institute of Management, SGH Warsaw School of Economics, Warsaw, Poland.

[2]Institute of Economic Sciences, University of Wroclaw, Wrocław, Poland.

[3]Institute of International Economic Policy, SGH Warsaw School of Economics, Warsaw, Poland.

[4]Department of Physical Education and Lifelong Sports, Poznan University of Physical Education, Poznań, Poland.

***Correspondence to:** Adrian Lubowiecki-Vikuk, Email: alubow@sgh.waw.pl

Citation: Lubowiecki-Vikuk A, Bartkowiak A, Biernat E, Kantanista A. Interest in weight loss methods among adults and its predictors: sociodemographic factors, anthropometric parameters, and physical activity. Int J Health Policy Manag. 2025;14:8493. doi:10.34172/ijhpm.8493

Supplementary file 1. Respondents' Characteristics (Including Tables S1-S6)

Table S1. Interest in Undertaking in Physical Activity

**Characteristic**	***N***	**Definitely not *N* = 37 (3.3%)**	**Rather not *N* = 185 (16.6%)**	**No opinion *N* = 172 (15.4%)**	**Rather yes *N* = 195 (17.5%)**	**Definitely yes *N* = 527 (47.2%)**
Gender	1,116					
Men		24 (65%)	76 (41%)	84 (49%)	70 (36%)	240 (46%)
Women		13 (35%)	109 (59%)	88 (51%)	125 (64%)	287 (54%)
Marital status	1,094					
Unmarried		17 (46%)	47 (26%)	68 (40%)	86 (45%)	244 (48%)
Married		20 (54%)	134 (74%)	102 (60%)	107 (55%)	269 (52%)
No answer (N/A)		0	4	2	2	14
Educational level	1,108					
Primary		8 (22%)	31 (17%)	15 (8.7%)	4 (2.1%)	4 (0.8%)
Vocational		24 (65%)	107 (59%)	109 (63%)	76 (39%)	132 (25%)
Secondary		3 (8.1%)	34 (19%)	39 (23%)	65 (34%)	195 (37%)
Higher		2 (5.4%)	10 (5.5%)	9 (5.2%)	49 (25%)	192 (37%)
N/A		0	3	0	1	4
Professional activity	1,116					
Professionals		1 (2.7%)	7 (3.8%)	9 (5.2%)	53 (27%)	173 (33%)

**Characteristic**	***N***	**Definitely not** ***N* = 37 (3.3%)**	**Rather not** ***N* = 185 (16.6%)**	**No opinion** ***N* = 172 (15.4%)**	**Rather yes** ***N* = 195 (17.5%)**	**Definitely yes** ***N* = 527 (47.2%)**
Technicians and associate professionals		0 (0%)	0 (0%)	3 (1.7%)	9 (4.6%)	27 (5.1%)
Service and sales workers		1 (2.7%)	11 (5.9%)	13 (7.6%)	30 (15%)	78 (15%)
Craft and related trades workers		1 (2.7%)	6 (3.2%)	18 (10%)	11 (5.6%)	34 (6.5%)
Plant and machine operators and assemblers		3 (8.1%)	19 (10%)	23 (13%)	16 (8.2%)	63 (12%)
Elementary occupations		0 (0%)	19 (10%)	14 (8.1%)	21 (11%)	21 (4.0%)
Non-employees		31 (84%)	123 (66%)	92 (53%)	55 (28%)	131 (25%)
Economic situation	1,107					
“Poor”		25 (68%)	74 (40%)	32 (19%)	11 (5.7%)	14 (2.7%)
“Hard to say”		6 (16%)	45 (25%)	69 (40%)	59 (30%)	91 (17%)
“Good”		6 (16%)	64 (35%)	70 (41%)	124 (64%)	417 (80%)
N/A		0	2	1	1	5
Level of physical activity	827					
Low		4 (33%)	20 (22%)	20 (19%)	25 (15%)	41 (9.0%)
Moderate		8 (67%)	50 (56%)	68 (66%)	84 (51%)	218 (48%)
Hight		0 (0%)	20 (22%)	15 (15%)	56 (34%)	198 (43%)
N/A		25	95	69	30	70
Age (classes)	1,116					
18–39		15 (41%)	43 (23%)	59 (34%)	103 (53%)	289 (55%)
40–59		6 (16%)	56 (30%)	49 (28%)	49 (25%)	164 (31%)
60+		16 (43%)	86 (46%)	64 (37%)	43 (22%)	74 (14%)
BMI status	1,116					
Underweight		0 (0%)	2 (1.1%)	3 (1.7%)	5 (2.6%)	45 (8.5%)
Normal		18 (49%)	47 (25%)	49 (28%)	83 (43%)	271 (51%)
Overweight		19 (51%)	136 (74%)	120 (70%)	107 (55%)	211 (40%)
WHR status	1,105					
Normal		34 (92%)	140 (77%)	150 (89%)	165 (86%)	467 (89%)
Abdominal obesity		3 (8.1%)	43 (23%)	19 (11%)	27 (14%)	57 (11%)
N/A		0	2	3	3	3
BF status	1,116					
Normal		12 (32%)	48 (26%)	56 (33%)	82 (42%)	309 (59%)

Characteristic	*N*	Definitely not *N* = 37 (3.3%)	Rather not *N* = 185 (16.6%)	No opinion *N* = 172 (15.4%)	Rather yes *N* = 195 (17.5%)	Definitely yes *N* = 527 (47.2%)
Obesity		25 (68%)	137 (74%)	116 (67%)	113 (58%)	218 (41%)

[1] n (%)

Table S2. Interest in Weight Loss Diet

Characteristic	*N*	Definitely not *N* = 39 (3.5%)	Rather not *N* = 199 (17.9%)	No opinion *N* = 198 (17.8%)	Rather yes *N* = 196 (17.6%)	Definitely yes *N* = 482 (43.2%)
Gender	1,114					
Men		28 (72%)	102 (51%)	106 (54%)	67 (34%)	189 (39%)
Women		11 (28%)	97 (49%)	92 (46%)	129 (66%)	293 (61%)
Marital status	1,092					
Unmarried		17 (44%)	62 (32%)	82 (42%)	80 (42%)	221 (47%)
Married		22 (56%)	134 (68%)	114 (58%)	112 (58%)	248 (53%)
No answer (N/A)		0	3	2	4	13
Educational level	1,106					
Primary		7 (18%)	29 (15%)	19 (9.6%)	2 (1.0%)	5 (1.0%)
Vocational		25 (64%)	118 (60%)	125 (63%)	74 (38%)	106 (22%)
Secondary		6 (15%)	44 (22%)	45 (23%)	65 (34%)	176 (37%)
Higher		1 (2.6%)	6 (3.0%)	9 (4.5%)	53 (27%)	191 (40%)
N/A		0	2	0	2	4
Professional activity	1,114					
Professionals		0 (0%)	4 (2.0%)	8 (4.0%)	56 (29%)	174 (36%)
Technicians and associate professionals		0 (0%)	0 (0%)	3 (1.5%)	11 (5.6%)	25 (5.2%)
Service and sales workers		1 (2.6%)	10 (5.0%)	15 (7.6%)	31 (16%)	75 (16%)
Craft and related trades workers		1 (2.6%)	10 (5.0%)	24 (12%)	13 (6.6%)	22 (4.6%)
Plant and machine operators and assemblers		3 (7.7%)	29 (15%)	30 (15%)	15 (7.7%)	47 (9.8%)
Elementary occupations		0 (0%)	20 (10%)	17 (8.6%)	20 (10%)	18 (3.7%)
Non-employees		34 (87%)	126 (63%)	101 (51%)	50 (26%)	121 (25%)
Economic situation	1,105					

Characteristic	*N*	Definitely not<br>*N* = 39 (3.5%)	Rather not<br>*N* = 199 (17.9%)	No opinion<br>*N* = 198 (17.8%)	Rather yes<br>*N* = 196 (17.6%)	Definitely yes<br>*N* = 482 (43.2%)
"Poor"		24 (62%)	74 (38%)	36 (18%)	9 (4.6%)	13 (2.7%)
"Hard to say"		9 (23%)	51 (26%)	72 (37%)	50 (26%)	89 (19%)
"Good"		6 (15%)	72 (37%)	89 (45%)	135 (70%)	376 (79%)
N/A		0	2	1	2	4
Level of physical activity	825					
Low		4 (31%)	23 (21%)	22 (17%)	24 (15%)	37 (9.0%)
Moderate		8 (62%)	59 (54%)	80 (63%)	79 (49%)	201 (49%)
Hight		1 (7.7%)	28 (25%)	25 (20%)	59 (36%)	175 (42%)
N/A		26	89	71	34	69
Age (classes)	1,114					
18–39		17 (44%)	60 (30%)	77 (39%)	99 (51%)	255 (53%)
40–59		6 (15%)	55 (28%)	53 (27%)	54 (28%)	155 (32%)
60+		16 (41%)	84 (42%)	68 (34%)	43 (22%)	72 (15%)
BMI status	1,114					
Underweight		0 (0%)	1 (0.5%)	4 (2.0%)	5 (2.6%)	45 (9.3%)
Normal		20 (51%)	61 (31%)	58 (29%)	88 (45%)	241 (50%)
Overweight		19 (49%)	137 (69%)	136 (69%)	103 (53%)	196 (41%)
WHR status	1,103					
Normal		35 (90%)	159 (81%)	171 (88%)	163 (84%)	426 (89%)
Abdominal obesity		4 (10%)	38 (19%)	23 (12%)	31 (16%)	53 (11%)
N/A		0	2	4	2	3
BF status	1,114					
Normal		14 (36%)	54 (27%)	63 (32%)	88 (45%)	287 (60%)
Obesity		25 (64%)	145 (73%)	135 (68%)	108 (55%)	195 (40%)

[1] n (%)

Table S3. Interest in Bariatric Surgery

Characteristic	*N*	Definitely not<br>*N* = 200 (18%)	Rather not<br>*N* = 351 (31.5%)	No opinion<br>*N* = 330 (29.7%)	Rather yes<br>*N* = 79 (7.1%)	Definitely yes<br>*N* = 153 (13.7%)
Gender	1,113					
Men		102 (51%)	177 (50%)	152 (46%)	15 (19%)	45 (29%)

Characteristic	*N*	Definitely not<br>*N* = 200 (18%)	Rather not<br>*N* = 351 (31.5%)	No opinion<br>*N* = 330 (29.7%)	Rather yes<br>*N* = 79 (7.1%)	Definitely yes<br>*N* = 153 (13.7%)
Women		98 (49%)	174 (50%)	178 (54%)	64 (81%)	108 (71%)
Marital status	1,091					
Unmarried		98 (50%)	126 (37%)	141 (43%)	34 (44%)	61 (41%)
Married		98 (50%)	216 (63%)	185 (57%)	44 (56%)	88 (59%)
No answer (N/A)		4	9	4	1	4
Educational level	1,105					
Primary		10 (5.1%)	31 (8.9%)	18 (5.5%)	1 (1.3%)	2 (1.3%)
Vocational		60 (30%)	162 (47%)	156 (48%)	28 (35%)	41 (27%)
Secondary		53 (27%)	93 (27%)	118 (36%)	24 (30%)	48 (32%)
Higher		75 (38%)	62 (18%)	36 (11%)	26 (33%)	61 (40%)
N/A		2	3	2	0	1
Professional activity	1,113					
Professionals		67 (34%)	54 (15%)	34 (10%)	26 (33%)	60 (39%)
Technicians and associate professionals		8 (4.0%)	7 (2.0%)	11 (3.3%)	5 (6.3%)	8 (5.2%)
Service and sales workers		17 (8.5%)	35 (10.0%)	42 (13%)	9 (11%)	29 (19%)
Craft and related trades workers		13 (6.5%)	20 (5.7%)	29 (8.8%)	4 (5.1%)	4 (2.6%)
Plant and machine operators and assemblers		20 (10%)	46 (13%)	47 (14%)	1 (1.3%)	10 (6.5%)
Elementary occupations		6 (3.0%)	31 (8.8%)	21 (6.4%)	8 (10%)	8 (5.2%)
Non-employees		69 (34%)	158 (45%)	146 (44%)	26 (33%)	34 (22%)
Economic situation	1,104					
"Poor"		32 (16%)	82 (23%)	39 (12%)	1 (1.3%)	2 (1.3%)
"Hard to say"		33 (17%)	81 (23%)	105 (32%)	23 (29%)	29 (19%)
"Good"		133 (67%)	186 (53%)	184 (56%)	54 (69%)	120 (79%)
N/A		2	2	2	1	2
Level of physical activity	824					
Low		13 (8.2%)	40 (16%)	39 (17%)	11 (17%)	7 (5.7%)
Moderate		76 (48%)	136 (56%)	133 (57%)	29 (45%)	53 (43%)
Hight		69 (44%)	68 (28%)	63 (27%)	25 (38%)	62 (51%)
N/A		42	107	95	14	31
Age (classes)	1,113					

Characteristic	*N*	Definitely not<br>***N* = 200 (18%)**	Rather not<br>***N* = 351 (31.5%)**	No opinion<br>***N* = 330 (29.7%)**	Rather yes<br>***N* = 79 (7.1%)**	Definitely yes<br>***N* = 153 (13.7%)**
18–39		103 (52%)	145 (41%)	144 (44%)	39 (49%)	77 (50%)
40–59		54 (27%)	106 (30%)	93 (28%)	25 (32%)	45 (29%)
60+		43 (22%)	100 (28%)	93 (28%)	15 (19%)	31 (20%)
BMI status	1,113					
Underweight		11 (5.5%)	17 (4.8%)	9 (2.7%)	2 (2.5%)	16 (10%)
Normal		120 (60%)	127 (36%)	125 (38%)	35 (44%)	59 (39%)
Overweight		69 (34%)	207 (59%)	196 (59%)	42 (53%)	78 (51%)
WHR status	1,103					
Normal		177 (88%)	291 (84%)	281 (86%)	69 (90%)	137 (90%)
Abdominal obesity		23 (12%)	56 (16%)	46 (14%)	8 (10%)	15 (9.9%)
N/A		0	4	3	2	1
BF status	1,113					
Normal		114 (57%)	140 (40%)	131 (40%)	37 (47%)	84 (55%)
Obesity		86 (43%)	211 (60%)	199 (60%)	42 (53%)	69 (45%)

[1] n (%)

Table S4. Interest in Liposuction

Characteristic	*N*	Definitely not<br>***N* = 183 (16.5%)**	Rather not<br>***N* = 339 (30.5%)**	No opinion<br>***N* = 310 (28%)**	Rather yes<br>***N* = 98 (8.8%)**	Definitely yes<br>***N* = 180 (16.2%)**
Gender	1,110					
Men		100 (55%)	172 (51%)	148 (48%)	20 (20%)	50 (28%)
Women		83 (45%)	167 (49%)	162 (52%)	78 (80%)	130 (72%)
Marital status	1,088					
Unmarried		91 (51%)	121 (37%)	134 (44%)	41 (43%)	70 (40%)
Married		89 (49%)	210 (63%)	173 (56%)	55 (57%)	104 (60%)
No answer (N/A)		3	8	3	2	6
Educational level	1,102					
Primary		9 (4.9%)	31 (9.2%)	19 (6.2%)	2 (2.0%)	1 (0.6%)
Vocational		57 (31%)	160 (48%)	148 (48%)	34 (35%)	48 (27%)
Secondary		49 (27%)	90 (27%)	109 (36%)	27 (28%)	58 (32%)
Higher		67 (37%)	55 (16%)	31 (10%)	35 (36%)	72 (40%)

Characteristic	*N*	**Definitely not**<br>***N* = 183 (16.5%)**	**Rather not**<br>***N* = 339 (30.5%)**	**No opinion**<br>***N* = 310 (28%)**	**Rather yes**<br>***N* = 98 (8.8%)**	**Definitely yes**<br>***N* = 180 (16.2%)**
N/A		1	3	3	0	1
Professional activity	1,110					
Professionals		60 (33%)	47 (14%)	29 (9.4%)	35 (36%)	71 (39%)
Technicians and associate professionals		6 (3.3%)	8 (2.4%)	10 (3.2%)	7 (7.1%)	8 (4.4%)
Service and sales workers		12 (6.6%)	32 (9.4%)	38 (12%)	10 (10%)	39 (22%)
Craft and related trades workers		13 (7.1%)	19 (5.6%)	29 (9.4%)	5 (5.1%)	4 (2.2%)
Plant and machine operators and assemblers		20 (11%)	45 (13%)	45 (15%)	3 (3.1%)	10 (5.6%)
Elementary occupations		6 (3.3%)	29 (8.6%)	20 (6.5%)	9 (9.2%)	11 (6.1%)
Non-employees		66 (36%)	159 (47%)	139 (45%)	29 (30%)	37 (21%)
Economic situation	1,101					
"Poor"		32 (18%)	82 (24%)	36 (12%)	4 (4.1%)	3 (1.7%)
"Hard to say"		28 (15%)	78 (23%)	99 (32%)	28 (29%)	35 (20%)
"Good"		121 (67%)	177 (53%)	173 (56%)	65 (67%)	140 (79%)
N/A		2	2	2	1	2
Level of physical activity	822					
Low		9 (6.3%)	38 (17%)	37 (17%)	13 (16%)	12 (8.1%)
Moderate		69 (49%)	127 (55%)	128 (58%)	40 (49%)	61 (41%)
Hight		64 (45%)	65 (28%)	55 (25%)	29 (35%)	75 (51%)
N/A		41	109	90	16	32
Age (classes)	1,110					
18–39		93 (51%)	137 (40%)	139 (45%)	48 (49%)	90 (50%)
40–59		49 (27%)	103 (30%)	83 (27%)	33 (34%)	56 (31%)
60+		41 (22%)	99 (29%)	88 (28%)	17 (17%)	34 (19%)
BMI status	1,110					
Underweight		11 (6.0%)	15 (4.4%)	9 (2.9%)	3 (3.1%)	17 (9.4%)
Normal		112 (61%)	118 (35%)	120 (39%)	47 (48%)	70 (39%)
Overweight		60 (33%)	206 (61%)	181 (58%)	48 (49%)	93 (52%)
WHR status	1,100					
Normal		163 (89%)	281 (84%)	269 (88%)	83 (86%)	156 (87%)
Abdominal obesity		20 (11%)	54 (16%)	38 (12%)	13 (14%)	23 (13%)

Characteristic	N	Definitely not N = 183 (16.5%)	Rather not N = 339 (30.5%)	No opinion N = 310 (28%)	Rather yes N = 98 (8.8%)	Definitely yes N = 180 (16.2%)
N/A		0	4	3	2	1
BF status	1,110					
Normal		108 (59%)	129 (38%)	124 (40%)	46 (47%)	99 (55%)
Obesity		75 (41%)	210 (62%)	186 (60%)	52 (53%)	81 (45%)

[1] n (%)

Table S5. Interest in Dietary Supplements to Support Weight Loss

Characteristic	N	Definitely not N = 70 (6.3%)	Rather not N = 218 (19.5%)	No opinion N = 237 (21.3%)	Rather yes N = 170 (15.2%)	Definitely yes N = 421 (37.7%)
Gender	1,116					
Men		51 (73%)	126 (58%)	127 (54%)	42 (25%)	146 (35%)
Women		19 (27%)	92 (42%)	110 (46%)	128 (75%)	275 (65%)
Marital status	1,094					
Unmarried		35 (50%)	75 (35%)	101 (43%)	82 (49%)	169 (42%)
Married		35 (50%)	140 (65%)	134 (57%)	87 (51%)	236 (58%)
No answer (N/A)		0	3	2	1	16
Educational level	1,108					
Primary		9 (13%)	27 (13%)	14 (5.9%)	2 (1.2%)	10 (2.4%)
Vocational		35 (50%)	111 (52%)	130 (55%)	47 (28%)	125 (30%)
Secondary		15 (21%)	53 (25%)	68 (29%)	59 (35%)	141 (34%)
Higher		11 (16%)	23 (11%)	25 (11%)	59 (35%)	144 (34%)
N/A		0	4	0	3	1
Professional activity	1,116					
Professionals		9 (13%)	19 (8.7%)	27 (11%)	56 (33%)	132 (31%)
Technicians and associate professionals		0 (0%)	2 (0.9%)	5 (2.1%)	10 (5.9%)	22 (5.2%)
Service and sales workers		1 (1.4%)	15 (6.9%)	19 (8.0%)	28 (16%)	70 (17%)
Craft and related trades workers		6 (8.6%)	12 (5.5%)	17 (7.2%)	10 (5.9%)	25 (5.9%)
Plant and machine operators and assemblers		10 (14%)	35 (16%)	46 (19%)	9 (5.3%)	23 (5.5%)
Elementary occupations		0 (0%)	16 (7.3%)	14 (5.9%)	10 (5.9%)	35 (8.3%)
Non-employees		44 (63%)	119 (55%)	109 (46%)	47 (28%)	114 (27%)

Characteristic	*N*	Definitely not *N* = 70 (6.3%)	Rather not *N* = 218 (19.5%)	No opinion *N* = 237 (21.3%)	Rather yes *N* = 170 (15.2%)	Definitely yes *N* = 421 (37.7%)
Economic situation	1,107					
“Poor”		26 (38%)	74 (34%)	29 (12%)	6 (3.6%)	22 (5.3%)
“Hard to say”		12 (18%)	39 (18%)	77 (33%)	39 (23%)	103 (25%)
“Good”		30 (44%)	103 (48%)	130 (55%)	124 (73%)	293 (70%)
N/A		2	2	1	1	3
Level of physical activity	827					
Low		5 (12%)	25 (19%)	26 (16%)	15 (11%)	39 (11%)
Moderate		21 (49%)	75 (56%)	105 (65%)	63 (45%)	164 (47%)
Hight		17 (40%)	35 (26%)	30 (19%)	63 (45%)	144 (41%)
N/A		27	83	76	29	74
Age (classes)	1,116					
18–39		32 (46%)	80 (37%)	108 (46%)	95 (56%)	194 (46%)
40–59		14 (20%)	59 (27%)	61 (26%)	46 (27%)	145 (34%)
60+		24 (34%)	79 (36%)	68 (29%)	29 (17%)	82 (19%)
BMI status	1,116					
Underweight		5 (7.1%)	6 (2.8%)	5 (2.1%)	7 (4.1%)	32 (7.6%)
Normal		38 (54%)	78 (36%)	90 (38%)	87 (51%)	174 (41%)
Overweight		27 (39%)	134 (61%)	142 (60%)	76 (45%)	215 (51%)
WHR status	1,105					
Normal		62 (89%)	185 (86%)	214 (91%)	149 (89%)	346 (83%)
Abdominal obesity		8 (11%)	31 (14%)	20 (8.5%)	19 (11%)	71 (17%)
N/A		0	2	3	2	4
BF status	1,116					
Normal		33 (47%)	76 (35%)	93 (39%)	90 (53%)	216 (51%)
Obesity		37 (53%)	142 (65%)	144 (61%)	80 (47%)	205 (49%)

[1] n (%)

Table S6. Interest in Weight Loss Drugs

**Characteristic**	***N***	**Definitely not** ***N*** **= 109 (9.7%)**	**Rather not** ***N*** **= 289 (25.8%)**	**No opinion** ***N*** **= 293 (26.2%)**	**Rather yes** ***N*** **= 122 (11%)**	**Definitely yes** ***N*** **= 305 (27.3%)**
Gender	1,118					
Men		67 (61%)	156 (54%)	150 (51%)	25 (20%)	96 (31%)
Women		42 (39%)	133 (46%)	143 (49%)	97 (80%)	209 (69%)
Marital status	1,096					
Unmarried		50 (47%)	106 (37%)	126 (43%)	57 (47%)	124 (42%)
Married		56 (53%)	178 (63%)	165 (57%)	64 (53%)	170 (58%)
No answer (N/A)		3	5	2	1	11
Educational level	1,110					
Primary		9 (8.3%)	29 (10%)	18 (6.1%)	1 (0.8%)	5 (1.6%)
Vocational		46 (42%)	136 (48%)	148 (51%)	34 (29%)	85 (28%)
Secondary		23 (21%)	76 (27%)	86 (29%)	44 (37%)	108 (36%)
Higher		31 (28%)	44 (15%)	41 (14%)	40 (34%)	106 (35%)
N/A		0	4	0	3	1
Professional activity	1,118					
Professionals		30 (28%)	39 (13%)	40 (14%)	37 (30%)	97 (32%)
Technicians and associate professionals		1 (0.9%)	5 (1.7%)	8 (2.7%)	8 (6.6%)	17 (5.6%)
Service and sales workers		6 (5.5%)	21 (7.3%)	29 (9.9%)	23 (19%)	54 (18%)
Craft and related trades workers		7 (6.4%)	16 (5.5%)	24 (8.2%)	5 (4.1%)	18 (5.9%)
Plant and machine operators and assemblers		12 (11%)	40 (14%)	57 (19%)	1 (0.8%)	14 (4.6%)
Elementary occupations		3 (2.8%)	24 (8.3%)	15 (5.1%)	8 (6.6%)	25 (8.2%)
Non-employees		50 (46%)	144 (50%)	120 (41%)	40 (33%)	80 (26%)
Economic situation	1,109					
“Poor”		30 (28%)	79 (28%)	35 (12%)	3 (2.5%)	10 (3.3%)
“Hard to say”		20 (19%)	60 (21%)	98 (34%)	28 (23%)	65 (21%)
“Good”		57 (53%)	148 (52%)	158 (54%)	90 (74%)	228 (75%)
N/A		2	2	2	1	2
Level of physical activity	829					
Low		9 (11%)	34 (17%)	32 (15%)	12 (12%)	23 (9.5%)
Moderate		32 (41%)	111 (55%)	131 (63%)	45 (45%)	110 (45%)

Characteristic	*N*	**Definitely not** *N* **= 109 (9.7%)**	**Rather not** *N* **= 289 (25.8%)**	**No opinion** *N* **= 293 (26.2%)**	**Rather yes** *N* **= 122 (11%)**	**Definitely yes** *N* **= 305 (27.3%)**
Hight		38 (48%)	56 (28%)	44 (21%)	42 (42%)	110 (45%)
N/A		30	88	86	23	62
Age (classes)	1,118					
18–39		52 (48%)	113 (39%)	134 (46%)	68 (56%)	143 (47%)
40–59		26 (24%)	87 (30%)	83 (28%)	29 (24%)	100 (33%)
60+		31 (28%)	89 (31%)	76 (26%)	25 (20%)	62 (20%)
BMI status	1,118					
Underweight		7 (6.4%)	9 (3.1%)	5 (1.7%)	5 (4.1%)	29 (9.5%)
Normal		62 (57%)	109 (38%)	115 (39%)	62 (51%)	121 (40%)
Overweight		40 (37%)	171 (59%)	173 (59%)	55 (45%)	155 (51%)
WHR status	1,107					
Normal		94 (86%)	238 (83%)	258 (89%)	109 (91%)	259 (86%)
Abdominal obesity		15 (14%)	48 (17%)	32 (11%)	11 (9.2%)	43 (14%)
N/A		0	3	3	2	3
BF status	1,118					
Normal		55 (50%)	108 (37%)	116 (40%)	67 (55%)	162 (53%)
Obesity		54 (50%)	181 (63%)	177 (60%)	55 (45%)	143 (47%)

[1] n (%)
