# Supplementary material for: Interest in Weight Loss Methods Among Adults and Its Predictors: Sociodemographic Factors, Anthropometric Parameters, and Physical Activity
Source: Int J Health Policy Manag. 2025 Jun 9;14:8493. doi: 10.34172/ijhpm.8493 (PMC12337211; doi:10.34172/ijhpm.8493)
Supplement: Supplementary file 2 — contains Figure S1. [file ijhpm-14-8493-s002.pdf]

Article title: Interest in Weight Loss Methods Among Adults and Its Predictors: Sociodemographic Factors, Anthropometric Parameters, and Physical Activity

Journal name: International Journal of Health Policy and Management (IJHPM)

Authors' information: Adrian Lubowiecki-Vikuk[1]*, Anna Bartkowiak[2], Elżbieta Biernat[3], Adam Kantanista[4]

[1]Institute of Management, SGH Warsaw School of Economics, Warsaw, Poland.

[2]Institute of Economic Sciences, University of Wroclaw, Wrocław, Poland.

[3]Institute of International Economic Policy, SGH Warsaw School of Economics, Warsaw, Poland.

[4]Department of Physical Education and Lifelong Sports, Poznan University of Physical Education, Poznań, Poland.

***Correspondence to:** Adrian Lubowiecki-Vikuk, Email: alubow@sgh.waw.pl

Citation: Lubowiecki-Vikuk A, Bartkowiak A, Biernat E, Kantanista A. Interest in weight loss methods among adults and its predictors: sociodemographic factors, anthropometric parameters, and physical activity. Int J Health Policy Manag. 2025;14:8493. doi:10.34172/ijhpm.8493

Supplementary file 2

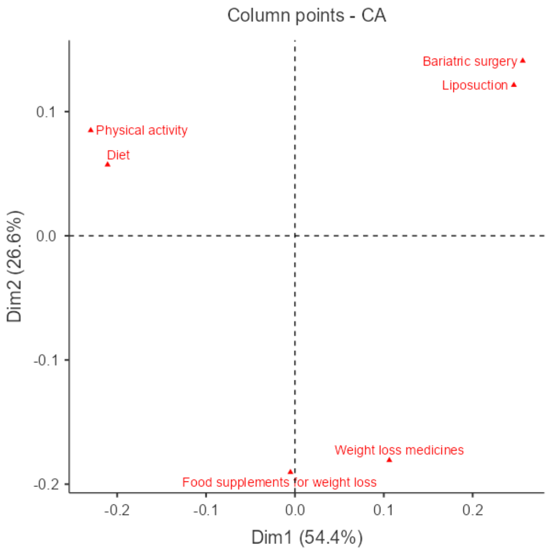

Fig. S1. Results from correspondence analysis for the analysed dependent variables.
