# Supplementary material for: Interest in Weight Loss Methods Among Adults and Its Predictors: Sociodemographic Factors, Anthropometric Parameters, and Physical Activity
Source: Int J Health Policy Manag. 2025 Jun 9;14:8493. doi: 10.34172/ijhpm.8493 (PMC12337211; doi:10.34172/ijhpm.8493)
Supplement: Supplementary file 3 — Results (including Tables S7-S14). [file ijhpm-14-8493-s003.pdf]

Article title: Interest in Weight Loss Methods Among Adults and Its Predictors: Sociodemographic Factors, Anthropometric Parameters, and Physical Activity

Journal name: International Journal of Health Policy and Management (IJHPM)

Authors' information: Adrian Lubowiecki-Vikuk[1]*, Anna Bartkowiak[2], Elżbieta Biernat[3], Adam Kantanista[4]

[1]Institute of Management, SGH Warsaw School of Economics, Warsaw, Poland.

[2]Institute of Economic Sciences, University of Wroclaw, Wrocław, Poland.

[3]Institute of International Economic Policy, SGH Warsaw School of Economics, Warsaw, Poland.

[4]Department of Physical Education and Lifelong Sports, Poznan University of Physical Education, Poznań, Poland.

***Correspondence to:** Adrian Lubowiecki-Vikuk, Email: alubow@sgh.waw.pl

Citation: Lubowiecki-Vikuk A, Bartkowiak A, Biernat E, Kantanista A. Interest in weight loss methods among adults and its predictors: sociodemographic factors, anthropometric parameters, and physical activity. Int J Health Policy Manag. 2025;14:8493. doi:10.34172/ijhpm.8493

Supplementary file 3. Results (Including Tables S7-S14)

Table S7. Correlations Between Dependent Variables and Predictors

	Physical activity	Weight loss diet	Bariatric surgery	Liposuction	Dietary supplements to support weight loss	Weight loss drugs
Level of physical activity*	**0.345 (p<.001)**	**0.282 (p<.001)**	0.023 (p=.446)	0.0248 (p=.411)	**0.121 (p<.001)**	**0.088 (p=.003)**
BMI status	**-0.126 (p<.001)**	*-0.085 (p=.002)*	**0.086 (p=.001)**	**0.069 (p=.009)**	0.008 (p=.777)	0.043 (p=.109)
BF status*	**-0.244 (p<.001)**	**-0.242 (p<.001)**	0.014 (p=.601)	0.000 (p=.997)	**-0.103 (p<.001)**	**-0.085 (p.002)**
WHR status*	**0.080 (p=.004)**	**0.057 (p=.038)**	0.024 (p=.386)	0.004 (p=.878)	**-0.052 (p=.046)**	0.023 (p=.399)
Age (classes)*	**-0.243 (p<.001)**	**-0.191 (p<.001)**	-0.009 (p=.714)	-0.030 (p=.245)	**-0.087 (p<.001)**	**-0.063 (p=.013)**
Economic situation*	**0.412 (p<.001)**	*0.402 (p<.001)*	**0.085 (p<.001)**	**0.113 (p<.001)**	**0.237 (p<.001)**	**0.212 (p<.001)**
Educational level*	*0.406 (p<.001)*	0.431 (p<.001)	0.040 (p=.115)	*0.080 (p=.002)*	**0.270 (p<.001)**	*0.194 (p<.001)*

Table S7. Correlations Between Dependent Variables and Predictors

	Physical activity	Weight loss diet	Bariatric surgery	Liposuction	Dietary supplements to support weight loss	Weight loss drugs
Professional activity[**]	*0.234 (p<.001)*	*0.257 (p<.001)*	**0.170 (p<.001)**	**0.190 (p<.001)**	*0.207 (p<.001)*	**0.183 (p<.001)**
Gender[**]	*0.114 (p=.006)*	*0.180 (p<.001)*	**0.198 (p<.001)**	**0.228 (p<.001)**	**0.284 (p<.001)**	**0.234 (p<.001)**
Marital status[**]	**0.156 (p<.001)**	**0.112 (p=.008)**	<u>0.092 (p=.056)</u>	**0.096 (p=.040)**	<u>0.092 (p=.053)</u>	<u>0.069 (p=.270)</u>

Notes: *Kendall's tau-b; **Cramer's V; variables with a weak link to the dependent variable were excluded from the analysis (underlined in the table). In some cases, there was a strong correlation between predictors, where the predictor with a weaker link to the dependent variable was excluded (in italics in the table).

Table S8. Significant and Insignificant Independent Variables for Each Analysed Dependent Variable from Ordinal Logistic Regression Analyses

Predictor	Physical activity	Weight loss diet	Bariatric surgery	Liposuction	Dietary supplements to support weight loss	Weight loss drugs
Level of physical activity[*]	+	+			-	-
BMI status	+	+	+	+		
BF status[*]	-	-			-	-
WHR status[*]	-	-			+	
Age (classes)[*]	+	+			+	-
Economic situation[*]	+		+	+	+	+
Educational level[*]		+			+	
Professional activity[**]			-	-		+
Gender[**]			+	+	+	+
Marital status[**]	-	-		-		

Notes: * Kendall's tau-b; **Cramer's V; + significant predictor; - insignificant variable, without sign variable excluded from analysis.

Table S9. Model Coefficients – Interest in Undertaking Physical Activity

Predictor	Estimate	SE	Z	p	Odds ratio	95% Confidence Interval	
						Lower	Upper
Level of physical activity							
Moderate – Low	0.377	0.207	1.82	0.068	1.458	0.971	2.183
Hight – Low	1.012	0.229	4.42	< .001	2.752	1.757	4.311
Age (classes)							
40–59 – 18–39	-0.206	0.170	-1.21	0.226	0.814	0.584	1.138
60+ – 18–39	-1.050	0.184	-5.72	< .001	0.350	0.244	0.501
Economic situation							
“Hard to say” – “Poor”	1.460	0.277	5.27	< .001	4.308	2.515	7.461
“Good” – “Poor”	2.334	0.267	8.74	< .001	10.320	6.148	17.540
BMI status							
Underweight – Normal	1.604	0.508	3.16	0.002	4.971	2.008	15.257
Overweight – Normal	-0.449	0.151	-2.97	0.003	0.638	0.474	0.858

Table 10. Model Coefficients – Interest in Weight Loss Diet

Predictor	Estimate	SE	Z	p	Odds ratio	95% Confidence Interval	
						Lower	Upper
Level of physical activity							
Moderate – Low	0.1423	0.207	0.689	0.491	1.153	0.768	1.727
Hight – Low	0.4732	0.226	2.097	0.036	1.605	1.030	2.498
Age (classes)							
40–59 – 18–39	0.0838	0.171	0.489	0.625	1.087	0.778	1.524
60+ – 18–39	-0.4685	0.183	-2.555	0.011	0.626	0.437	0.897
Economic situation							
“Hard to say” – “Poor”	1.2876	0.286	4.497	< .001	3.624	2.077	6.393
“Good” – “Poor”	1.5461	0.288	5.375	< .001	4.693	2.682	8.298
BMI status							
Underweight – Normal	1.5592	0.479	3.256	0.001	4.755	1.998	13.416
Overweight – Normal	0.2184	0.215	1.015	0.310	1.244	0.817	1.901

Table 10. Model Coefficients – Interest in Weight Loss Diet

Predictor	Estimate	SE	Z	p	Odds ratio	95% Confidence Interval	
						Lower	Upper
Educational level							
Vocational – Primary	0.1865	0.378	0.493	0.622	1.205	0.573	2.537
Secondary – Primary	1.0556	0.405	2.606	0.009	2.874	1.296	6.379
Higher – Primary	1.8216	0.425	4.283	< .001	6.182	2.684	14.283
BF status							
Obesity – Normal	-0.5339	0.218	-2.446	0.014	0.586	0.382	0.899

Table S11. Model Coefficients – Interest in Bariatric Surgery

Predictor	Estimate	SE	Z	p	Odds ratio	95% Confidence Interval	
						Lower	Upper
Economic situation							
"Hard to say" – "Poor"	0.986	0.174	5.658	< .001	2.68	1.907	3.78
"Good" – "Poor"	1.031	0.159	6.489	< .001	2.80	2.056	3.83
Gender							
Women – Men	0.597	0.112	5.322	< .001	1.82	1.459	2.26
BMI status							
Underweight – Normal	0.255	0.280	0.913	0.361	1.29	0.746	2.24
Overweight – Normal	0.387	0.117	3.303	< .001	1.47	1.171	1.85

Table S12. Model Coefficients – Interest in Liposuction

Predictor	Estimate	SE	Z	p	Odds ratio	95% Confidence Interval	
						Lower	Upper
Gender							
Women – Men	0.754	0.113	6.687	< .001	2.13	1.705	2.65
Economic situation							
"Hard to say" – "Poor"	1.069	0.175	6.113	< .001	2.91	2.070	4.11
"Good" – "Poor"	1.156	0.160	7.227	< .001	3.18	2.325	4.35
BMI status							
Underweight – Normal	0.178	0.279	0.640	0.522	1.20	0.692	2.07

Table S12. Model Coefficients – Interest in Liposuction

Predictor	Estimate	SE	Z	p	Odds ratio	95% Confidence Interval	
						Lower	Upper
Overweight – Normal	0.302	0.117	2.583	0.010	1.35	1.076	1.70

Table S13. Model Coefficients – Interest in Dietary Supplements to Support Weight Loss

Predictor	Estimate	SE	Z	p	Odds ratio	95% Confidence Interval	
						Lower	Upper
Gender							
Women – Men	0.913	0.117	7.794	< .001	2.491	1.981	3.14
Economic situation							
"Hard to say" – "Poor"	1.641	0.197	8.327	< .001	5.160	3.514	7.61
"Good" – "Poor"	1.502	0.195	7.689	< .001	4.490	3.067	6.60
WHR status							
Abdominal obesity – Normal	0.413	0.176	2.341	0.019	1.511	1.072	2.14
Age (classes)							
40–59 – 18–39	0.317	0.136	2.333	0.020	1.372	1.053	1.79
60+ – 18–39	-0.129	0.142	-0.904	0.366	0.879	0.665	1.16
Educational level							
Vocational – Primary	0.575	0.261	2.208	0.027	1.777	1.065	2.96
Secondary – Primary	1.077	0.282	3.815	< .001	2.937	1.687	5.11
Higher – Primary	1.528	0.303	5.043	< .001	4.609	2.544	8.35

Table S14. Model Coefficients – Interest in Weight Loss Drugs

Predictor	Estimate	SE	Z	p	Odds ratio	95% Confidence Interval	
						Lower	Upper
Gender							
Women – Men	0.838	0.130	6.450	< .001	2.313	1.794	2.987
Professional activity							
P2 – P1	0.437	0.318	1.375	0.169	1.549	0.834	2.916
P3 – P1	0.323	0.204	1.579	0.114	1.381	0.926	2.063
P4 – P1	0.167	0.273	0.614	0.539	1.182	0.693	2.021
P5 – P1	-0.528	0.217	-2.428	0.015	0.590	0.385	0.903
P6 – P1	-0.210	0.251	-0.837	0.403	0.810	0.495	1.327

Table S14. Model Coefficients – Interest in Weight Loss Drugs

						95% Confidence Interval	
Predictor	Estimate	SE	Z	p	Odds ratio	Lower	Upper
P7 – P1	-0.248	0.169	-1.464	0.143	0.780	0.560	1.088
Economic situation							
"Hard to say" – "Poor"	1.330	0.188	7.084	< .001	3.780	2.620	5.470
"Good" – "Poor"	1.638	0.183	8.963	< .001	5.145	3.602	7.376

Notes: Professional activity: P1 – professionals; P2 – technicians and associate professionals; P3 – service and sales workers; P4 – craft and related trades workers; P5 – plant and machine operators and assemblers; P6 – elementary occupations; P7 – non-employees.
